# Impact of a Food Rebalancing Program Associated with Plant-Derived Food Supplements on the Biometric, Behavioral, and Biological Parameters of Obese Subjects

**DOI:** 10.3390/nu15224780

**Published:** 2023-11-14

**Authors:** Jean-Jacques Houben, Yvon Carpentier, Genevieve Paulissen, Georges Van Snick, Antoine Soetewey

**Affiliations:** 1 Department of Digestive Surgery, Clinic of Metabolic Surgery, Centre Hospitalier Régional Sambre Meuse, Namur and Free University of Brussels (Université Libre de Bruxelles), Rue Chère-Voie 75, B 5060 Sambreville, Belgium; 2 Nutrition Lipid Development, Free University of Brussels (Université Libre de Bruxelles), Av. Octave Michot 17, 1640 Rhode Saint Genèse, Belgium; 3 Laboratoire SYNLAB, Biologie Clinique et Nutritionnelle, Av. Alexandre Fleming 3, 6220 Heppignies, Belgium; genevieve.paulissen@synlab.be; 4 Institut Européen de Physionutrition et de Phytothérapie, 12/14 Rond-Point des Champs Elysées, 75008 Paris, France; ipi@skynet.be; 5 Institute of Statistics, Biostatistics and Actuarial Sciences, Université Catholique de Louvain, 1348 Louvain-la-Neuve, Belgium; antoine.soetewey@uclouvain.be

**Keywords:** obesity, plant derivative, nutritional behavior, insulin resistance, lipid profile, cardiovascular risk, coaching, food supplement

## Abstract

Between 2021 and 2023, the Scientific Council of Dietplus^®^, a group specialized in overweight and obesity management, conducted a clinical study on 170 volunteer subjects with a BMI > 29 Kg/m^2^ consecutively recruited. The Dietplus^®^ program comprises nutritional education, intensive, personalized coaching, and consuming food supplements rich in plant derivatives. The aim of this study was to assess the effect of the Dietplus^®^ program on biometric, behavioral, and biological parameters. A control group of 30 obese patients was followed for a similar 12-week period. Mean weight loss reached 9 ± 2.1 kg in the Dietplus^®^ test group versus a 1 ± 0.1 kg weight gain in the control group. Excess weight loss reached 33 ± 13%, and fat mass loss was 7.6% (*p* < 0.001); waist circumference was reduced by 30%. Quality of Life, Nutriscore, and Prochaska di Clemente scale significantly improved (*p* < 0.001). Biological parameters showed substantial improvements in the carbohydrate profile and insulin resistance (HOMA index) and in the lipid profile with lower plasma triglyceride (*p* < 0.01) and VLDL (*p* < 0.01) concentrations. Inflammatory parameters (orosomucoid, ultrasensitive C-reactive protein, and PINI indices) were also substantially reduced. These results indicate a substantial benefit in subjects who followed the Dietplus^®^ program. (Dietplus^®^ 116 Rue Robert Bunsen, 57460 Behren-lès-Forbach, France is active in France Belgium and Spain. Plant Derived Food Supplements are produced in France). Indeed, improvements were observed in all biometric, behavioral, and metabolic parameters.

## 1. Introduction

The morbidity and excess mortality induced by obesity, as defined by the body mass index (BMI), are of considerable concern [1,2]. In Europe, the prevalence of overweight and obesity is 31.9% and 13.9%, respectively [3], lower than in the USA (~40% and 22%, respectively). 

While general practitioners focus primarily on prevention [4], pharmaceutical companies are developing new incretins [5,6], and surgeons are applying bariatric procedures to fight morbid obesity [7,8], several companies have proposed varied empirical and sometimes controversial dietary approaches [9,10,11]. More recently, modification of the gut microbiota has shown substantial benefits in overweight and obese patients [12]. 

Over the last ten years, the Dietplus^®^ program has focused on the nutritional management of obesity (30 > BMI > 35) and overweight (25 < BMI < 30) without invasive procedures. This concept is based on personalized assessment, coaching, and follow-up. Food supplements validated by Belgian and European legislations [13,14] are compiled, and diets, food products, and personalized recipes and dishes are recommended. 

The literature provides poor objective evidence on the effect of nutritional programs alone or in combination with the recommended phyto-derived food supplements (PDFSs) to facilitate dietary rebalancing [15,16]. Nevertheless, the benefits of human clinical trials using PDFSs have been reported [17,18,19,20,21,22,23,24,25], including improved well-being, loss of body weight, better lipid profile, fewer osteoarticular complaints, lower blood pressure, lower glycemic levels, and reduced respiratory insufficiency and cardiovascular risk. Some of these factors are directly associated with weight control and improved cholesterol profiles [26]. 

This study aims to assess the morphometric, behavioral, quality of life, and biological effects of a dietary rebalancing program, including potentiation with PDFSs. 

## 2. Materials and Methods

### 2.1. Study Population, Inclusion Criteria and Enrollment

One hundred and seventy obese subjects (BMI > 29) were consecutively enrolled in the Dietplus^®^ program for a period of 12 weeks on a voluntary basis. The participants agreed to adhere to the weight-control program and participate in the clinical study, including close anthropometric, behavioral, and biological evaluation. Medical history recorded prior to the Dietplus^®^ treatment showed that only 39% of the subjects reported no medical pathology of a metabolic nature. Meanwhile, 61% suffered from associated pathologies: treated hypertension (22), disabling osteoarticular and back pathologies (10), dyslipidemia (5), treated diabetes (4), gastroesophageal reflux (3), cardiorespiratory insufficiency (1), non-alcoholic steatohepatitis (1) (NASH), and substituted hypothyroidism unrelated to obesity (8). Several obesity-related malignant neoplasms were reported, including two surgically resected breast cancers.

Simultaneously, 30 obese control subjects were recruited voluntarily during consultations with a multidisciplinary medical team for a medical work-up. These participants did not undergo therapeutic intervention or take medication during observation. They were also assessed for motivation, morphometric, and blood variations and underwent ultrasound, cardiac workup, sleep polysomnography, gastroscopy, and preoperative radiological examinations, and preoperative assessment. Furthermore, they received information about their medical condition without any therapeutic interference. None of the controls took PDFSs, nor were dietary restrictions imposed. Due to the hazards associated with the COVID-19 pandemic and the lack of knowledge regarding the drop-out rate, the study period was extended [27].

### 2.2. The Dietplus^®^ Program 

After a parametric and nutritional assessments and the preventive history, the Dietplus^®^ program implements nutritional education on a low-calorie diet, personalized coaching on an adequate way of life, and regular intake of PDFS. 

The nutritional educational aspect includes oral and written training in nutrition and diet, covering the basics of a balanced diet comprising proteins, carbohydrates, and lipids. Meal collection, preparation, cooking, and presentation are then reviewed. Personalized coaches are assigned to each subject for the duration of the program. These coaches provide weekly guidance to boost motivation and alter eating and sleeping patterns. The subject commits to following the complete plan, which is re-evaluated every week in accordance with the regulations in each country (monitored by an independent subcontractor: https://www.pharmanager-development.com/ (accessed on 15 May 2023)). The weekly administration of the PDFS associations is applied in the same way for each subject. The information regarding the properties follows the PDFS Belgian regulations [28]. European and national Institutions regulating the PDFS determine which claimed properties can be mentioned.

The PDFS program is changed every week. The content and the sequence of PDFS associations are classified into 3 groups: PDFSs directly linked to weight loss, providing satiety, and reducing appetite; PDFSs and food supplements for digestion and sleep; and food plans specifically developed for their dietary properties. The latter contains organic cereals based on wheat germ and various products with a low glycemic index and a high protein content. The PDFSs provided in the global program include green tea leaf, ash leaf, fennel seed, cinnamon bark, dandelion leaf, quack grass rhizome, orthosiphon leaf, black elderberry, organic rosemary leaf, elderflower, green tea dry extract, turmeric root, dandelion leaf, nopal powder, chitosan, *Ascophyllum thallus*, artichoke leaf, dry extract of green mate, artichoke dry extract, cola nut, dry extract of guarana, L-carnitine, chromium chloride, marshmallow leaf, oat seed, mate dry extract, guarana seed dry extract, brewer’s yeast powder, black radish root powder, vitamin B6, chromium chloride, caffeine, fructooligosaccharides, *Fucus thallus*, oat grain, konjac root, griffonna seed, and a multivitamin supplement. The composition of the 9 consecutive weekly programs is detailed in the Supplementary Materials (www.gastrospace.com/nutrients/, accessed on 15 May 2023). 

### 2.3. Exclusion Criteria, Dropout, and Protocol Discontinuation

Exclusion criteria include a previous history of bariatric surgery, insulin-dependent diabetes, inflammatory bowel disease, neuropsychiatric pathology, or corticosteroid therapy. Furthermore, opposition from the attending physician made enrollment impossible. Subjects were considered to have dropped out if they refused to continue with the study protocol or if they deviated from the Dietplus^®^ program.

The protocol anticipated three obstacles: refusal to cope with the burdensome follow-up coaching, the demands of the study protocol (digital anamnesis, blood sampling, repeated measurements), and the prolonged impact on mobility due to the COVID-19 pandemic. 

### 2.4. Medical and Anthropometric Evaluation

Addiction to smoking or/and alcoholism was assessed. Medication (antihypertensive drugs, non-steroid anti-inflammatory drugs, hormonal substitutes, etc.) was maintained. Anthropometric measurements included age, height (cm), weight (kg), BMI (kg/m^2^), waist circumference (cm) (Pointer > 88 in women and > 102 in men) [29], heart rate (2 readings), and blood pressure (mmHg). Body composition was estimated by impedancemetry with inBody^®^270 (inBody Co. Ltd. Gangnam-gu, Seoul, Korea) [30,31].

### 2.5. Biological Evaluation

All biochemical tests were performed by the same laboratory (SYNLAB Heppignies, accredited laboratory under number 85261020) and included hemoglobin, hematocrit, erythrocytes, VCM, TIBC, serum iron, hydromineral balance, blood ionogram (Na^+^/K^+^/Cl^−^/HCO^3−^), Vit D, Mg++, phosphorus, chronic inflammatory state (CRP US, orosomucoid, fibrinogen), PINI index (prognostic inflammatory and nutritional index) [32], nutritional status (total protein level, albuminemia, prealbuminemia), and renal function (urea, creatinine, uric acid). Resistance to insulin and potential diabetes was assessed by fasting blood sugar, insulinemia—homeostatic model assessment of insulin resistance (HOMA) [33], and quantitative insulin sensitivity check index (QUICKI-index) [34]. QUICKI = 1/[log(I_0_) + log(G_0_)], where I_0_ is the fasting insulin (μU/mL), and G_0_ represents the fasting glucose (mg/dL). Lipid parameters included total cholesterol, triglycerides, LDL, VLDL, non-LDL, and HDL cholesterol. Cardiovascular risk (apolipoproteins B, Apolipo A/B ratio, atherosclerotic index) and liver function (PAL, GGT) were also assessed.

### 2.6. Behavioral Evaluation and Monitoring

Evaluation of the psycho-affective state, motivation, determination, and affective environment was evaluated by several self-assessment scales completed online by each subject. A WHO-compliant “quality of life” scale was applied before and after the protocol. Prochaska and Di Clemente developed a scale based on multiple contradictory questions [35,36] to test the subject’s ability to change. The study focused on lifestyle changes, such as consumption habits, eating habits, physical activity, and, more generally, lifestyle changes (Supplementary Materials).

The Mac Gill scale [37] validated for obese patients [38] was applied. The success of any weight control program depends on the attitude toward food, particularly the organization of meals, food preparation, and “conscientiousness” [39,40,41,42]. Indeed, obesity alters taste, and relative anosmia favors obesity [43,44]. The subjects were submitted to a 20-item nutritional questionnaire, “Nutriscore.” The score, calculated on a scale of 60, bears no relation to the one used in food chain labeling [45] (Supplementary Materials).

Daily physical activity was assessed using a 15-question online questionnaire focusing on four areas of daily life: private and professional spheres, leisure, and structured sports activity (Supplementary Materials). Only 74 subjects who responded to all four online questionnaires were considered for data collection.

### 2.7. Statistical Analysis 

Statistical analysis was performed using R Statistical Software (v4.2.2; R Core Team 2022). Data were presented as median (range) or mean ± standard deviation (SD) for continuous variables according to non-normal or normal distribution (determined by the Shapiro–Wilk test), respectively. For categorical variables, data were presented as numbers (percentages), whereas continuous variables, pre- and post-treatment data were compared using a Wilcoxon signed-rank test for non-normal distributions or a Student’s *t*-test for paired samples with normal distribution. The threshold for statistical significance was set at *p* < 0.05. Effect sizes were calculated as follows: Cohen’s d (1988) for parametric tests (small effect: d ≥ 0.2; mean effect: d ≥ 0.5; large effect: d ≥ 0.8 and below 0.2 is trivial), and rank-biserial correlation for non-parametric tests.

## 3. Results

**Figure 1:**
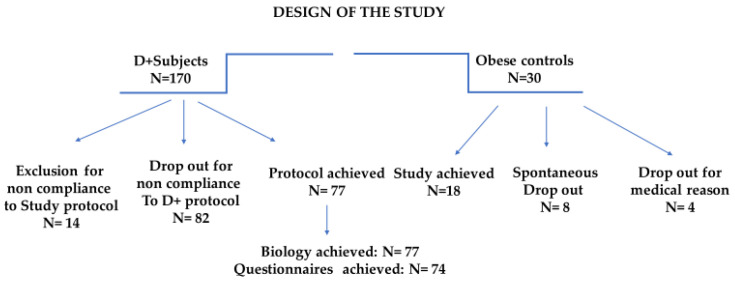
Study design focusing on the clinical pathways and incidence of dropout and exclusion.

The program compliance of the 200 subjects is illustrated in Figure 1.

### 3.1. Dropout: Analysis and Interpretation 

The protocol interruptions in Dietplus^®^ and control subjects are presented for both groups. Of the 30 controls, 5 did not follow the clinical itinerary, and 4 had a pathology contraindicating the continuation of the bariatric surgery protocol (neuroendocrine tumor, pregnancy, acute diverticulitis, hepatic mass). Finally, family constraints, financial issues, and a lack of compliance with the study protocol impacted one patient each. 

Protocol interruptions for Dietplus^®^ and control subjects were comparable. The investigators called back the 95 subjects excluded or lost to follow-up; 15 could not be contacted or refused to be interviewed. Meanwhile, 80 explained their failure, revealing five major causes and four more anecdotal causes. (Table 1) 

Among Dietplus^®^ subjects, the five main reasons for abandonment include a severe lack of compliance with the Dietplus^®^ program (26.2%), medical exclusion criteria (15%), family constraints with the predominance of strong opposition from a life partner, financial constraints (weekly cost was estimated between 40 and 50 €) and logistical problems often related to professional constraints. 

### 3.2. Comparison of the Two Cohorts 

We sought to determine whether the profile of control patients differed from that of the Dietplus^®^ customers. The profiles were similar in age, sex, BMI, and abdominal obesity. Nevertheless, the Dietplus^®^ subjects dropped out more often, had fewer co-morbidities, and a lower weight-loss target (−19 kg) than the control group (−28 kg). (Table 2)

At the family, behavioral, and psychological levels, the two cohorts were comparable, with even alcohol consumption and smoking identical. Moreover, upon enrollment, the Dietplus^®^ subjects had an average height of 165.1 ± 11 cm and mean weight of 94.1 ± 14.2 kg, with extremes ranging from 73 to 158 kg. Regarding abdominal obesity, the waist circumference in the 148 women was 108.4 ± 12 cm, categorized as pathological for 77 subjects.

The two cohorts were compared at the time of enrollment regarding psycho-behavioral scores (Table 3). Notably, control patients were referred for medical treatment, whereas Dietplus^®^ subjects sought a non-medical approach. While the quality of life and sporting activities were identical in both cohorts, dietary and nutritional behavior was significantly (*p* < 0.01) more disturbed in Dietplus^®^ subjects, with a nutriscore of 43.5 ± 4.2% versus 59 ± 5.7%. However, the ability to change (Prochaska Di Clemente Scale) was significantly improved (*p* < 0.01) when the Dietplus^®^ program was considered.

The two cohorts presented reasonably comparable profiles, with higher expectations in obese patients consulting a medical team. Eating habits were more compromised in subjects within the Dietplus^®^ group.

### 3.3. Evolution of the Control Group

The slight weight gain observed in the control group expected as obese patients are more likely to seek medical attention during periods of weight regain. During the work-up and observation periods, the weight gain was 1.0 ± 0.2 kg. The results were expected given that no therapeutic action was applied in the controls. However, the medical and paramedical consultations led to “awareness” as ¼ of patients moved from the contemplation stage to the determination stage.

The 12 weeks of medical observation and communication of the results of the medical work-up did not improve quality of life, nutritional habits, or physical activity. The only observed impact of the medical work-up was positive changes in motivation. (Table 4) 

### 3.4. Effect of The Dietplus^®^ Cure 

The effect of the program was measured via pre- and post-therapeutic values. Nevertheless, the comparison with the control group was useful as the natural evolution of obesity was unfavorable during the same period. For example, at the end of the Dietplus^®^ program, subjects lost an average of 10 ± 2.2 kg compared to the control group whose overweight increased. 

#### 3.4.1. Anthropometric and Hemodynamic Effects

The absolute weight loss of the Dietplus^®^ population was 9 ± 2.1 kg in 12 weeks. Excess weight expressed as a percentage was 38 ± 18% before the Dietplus^®^ program. After 12 weeks, it was 25 ± 17%, representing a more than 1/3 overweight loss. The loss was primarily abdominal (morbid obesity). No correlation was found between weight loss and age. 

The average abdominal circumference of the 22 men was 122 ± 12.5 cm, systematically (100%) above the tolerable limit (106 cm). The clinical measurement of the abdominal perimeter at the umbilicus level was reproducible, dropping by 8.3 ± 2.7 cm (i.e., a 29% loss of abdominal obesity; *p* < 0.001). (Table 5)

The weight loss was strictly limited to the fat mass with no negative impact induced by the program (PDFS included) on water or muscle compartments. 

The 77 compliant subjects treated during the 12-week protocol had virtually the same characteristics as all subjects in the study: mean starting weight of 95.7 ± 16 kg; pathological abdominal circumference in 100% of cases, averaging 110.6 ± 12.7 cm in women and 119 ± 9.5 cm in men; mean heart rate was 76.1 ± 10.1 beats/min. 

Heart rate measurements (at the beginning and end of the examination) showed an average of 76.7 ± 11.4 beats/min. Eleven subjects had a heart rate < 60 beats/min (relative bradycardia), and 3 had repetitive tachycardia > 100 beats/min.

Normal blood pressure values were interpreted according to international recommendations (i.e., < 140 mmHg systolic and < 90 mmHg diastolic). At enrollment, we identified 7 cases of isolated systolic hypertension, 23 cases of diastolic hypertension, and 33 cases of double-measured combined systolic and diastolic hypertension. Of these 63 abnormal measurements, only 43 (68%) had reported background hypertensive treatment. Suspected unrecognized hypertension was thus identified in 32%, or 11% of the enrolled subjects. The mean pretherapeutic blood pressure was 130.6/85.2 mmHg.

Fifteen subjects had two measurements of systolic–diastolic hypertension, six had confirmed diastolic hypertension and five had isolated systolic hypertension; only eight were aware of their condition. The clinical study revealed 24% of suspected untreated hypertension. At the end of the protocol, the average systolic blood pressure was 127.7/81.8 mmHg compared to 130.6/85.2 before implementation of the Dietplus^®^ program; 9/26 subjects (35%) stopped showing signs of arterial hypertension (*p* < 0.02). 

#### 3.4.2. Behavioral Parameters

The behavioral effects of the Dietplus^®^ program are independent of biological changes.

▪ Quality of Life

The QOL was 33.8 ± 6.1 /60 at the start of treatment and reached 37.4 ± 4.8 after 12 weeks (*p* < 0.001). At the time of enrollment, the negative impact of overweight altered the feeling of improved health. Fatigue was the main handicap, without affecting the ability to drive a car or perform daily household tasks. 

After the Dietplus^®^ program and subsequent weight loss, QOL improved in all areas of well-being, but most significantly in perceived overall health, sleep quality and, consequently, fatigue and mobility.

▪ Nutriscore

At enrollment, the questionnaire resulted in a Nutriscore of 43.3 ± 4.5/100. All nutritional principles were impacted (food volume, knowledge of nutritional principles, screen addiction). After 12 weeks of workup, the Nutriscore reached 45.9 ± 3.9 (*p* < 0.001). Improvements primarily concerned snacking, menu planning, water consumption, reduced carbonated drinks, soft drink consumption, volumes ingested, and screen addiction during meals. Unfortunately, and curiously, fruit and vegetable consumption did not change significantly. Despite the improvement, the Nutriscore did not explain such a significant weight loss.

▪ Physical Activity

Even if physical training is not incorporated into the program, sport is encouraged. The initial score upon enrolment of 39.3 ± 10.1 reached 43.0 ± 9.7 by the end of the study. The improvement was primarily due to physical activity during free time (*p* < 0.005) and the start of a structured sports activity.

▪ Prochaska and Di Clemente scale

Given the requirements of the online questionnaire, 74 subjects were properly scored. After requalifying the profile of each subject in the different postures (precontemplation, contemplation, determination, and action), contingency tables were drawn to compare the samples with each other and the treated cohort before and after the Dietplus^®^ program. The Prochaska Di Clemente scale was reproducible and homogeneous among the cohorts studied. The obese control subjects and those enrolled in Dietplus^®^ had very low motivation scores at the outset.

There was no difference between the obese control patients consulting a medical center and subjects entering the Dietplus^®^ program. Moreover, there was no difference in motivation between those who dropped out and those who completed the protocol and the study. It is impossible to predict the adhesion and compliance with weight control with the Prochaska Di Clemente scale. The effect of the Dietplus^®^ program, namely the coaching, on motivation was apparent (*p* < 0.001). (Table 6)

#### 3.4.3. Biological and Metabolic Effects

No significant difference in biological values was observed in the control group over the 12-week observation period. The values at the end of the protocol provided a frame of reference for the changes observed after the Dietplus^®^ program.

▪ Hematology and protein metabolism

No impact was observed on hematological parameters or serum protein and albumin levels. Prealbumin levels decreased after 12 weeks (*p* < 0.01).

▪ Hydromineral metabolism

No change in hydromineral metabolism was observed.

▪ Liver enzymes

No significant change occurred in cytolytic or cholestatic enzymes. however, the gamma-glutamyl transferase level (GGT), which reflects non-alcoholic steatohepatitis (NASH), was reduced from 35.4 ± 42.7 to 25.1 ± 15.0 after the Dietplus^®^ program (*p* < 0.001). The interpretation of GGT values was considered in relation to sex and age; 11/25 patients (44%) normalized their GGT level (*p* < 0.05) over the 12 weeks.

▪ Renal function

The plasma levels of urea and uric acid remained stable. The median value of creatinine concentration appeared to be significantly increased. However, considering the number of subjects presenting a plasma level > 1 mg/dL (10 before the cure and 11 after the 12 weeks), no renal insufficiency appeared.

▪ Glucose metabolism

Insulin resistance and type II diabetes were commonly observed over the study period. That is, 7/77 (10%) showed an increased fasting blood glucose at the onset, while only two (29%) returned to normal levels after 12 weeks. (Table 7)

A high HOMA index was measured in 26/49 subjects at the onset of the study and in only 11/49 at the end (*p* < 0.001). A similar trend was observed for the QUICKI index (*p* < 0.01).

▪ Lipid metabolism

The Dietplus^®^ program induced a significant decrease in many lipid components, particularly in the total, LDL, and VLDL cholesterol fractions. Normalization of the plasma levels appeared in 21/40 (50%) subjects with high total cholesterol levels, 10/16 (63%) with triglycerides, 13/42 (31%) with LDL, and 11/14 (79%) with VLDL. (Table 8)

▪ Inflammatory syndrome

The benefit of the cure on chronic inflammatory syndrome associated with obesity was obvious. When the CRP level was significantly increased, usCRP was considered not interpretable. (Table 9)

▪ Vitamin D

The level of 25OH Vit D improved significantly over the 12 weeks from 26.4 ± 10.0 µg/L to 31.5 ± 13.3. µg/L (*p* < 0.001). More specifically, 70/77 subjects (90%) were deficient (tolerance threshold in Belgium is > 50 ug/L); 12/70 (17%) completely corrected their deficiency; 44/77 (57%) improved their levels significantly, without normalizing them; 21/77 subjects (27%) showed a worsening of their 25-OH-vitamin D deficiency. Most subjects completed their treatment at the end of the winter. The overall improvement remained highly significant.

#### 3.4.4. Late Morphometric Measurements (24 Weeks)

Seventy-five patients who reached 24 weeks of follow-up (12 weeks after the end of the program) were called back for weight and impedance measurements; 46 accepted. Body weight loss remained stabilized at −9.9 ± 2.7 kg, and body fat at −33.4 ± 10.1. Most (56%) of subjects maintained these results six months later, and many others (35%) continued to improve.

## 4. Discussion

This study exhibited a major drop-out rate in both study groups. Hence, helping obese individuals control their diet and energy expenditure remains a major challenge [46,47,48,49].

### 4.1. Common Clinical Problems Observed in Obese Subjects

In the control population, almost one-third of patients dropped out during the development process. In this instance, the phenomenon of medical shopping is emphasized as obese patients often consider being overweight as a cosmetic problem [50]. Such a high drop-out rate was observed in both cohorts. This underscores the difficulty in motivating changes in dietary behavior and incorporating physical activity. 

The certainty of knowing one’s solution to the problem further complicates the relationship with any therapist. Moreover, the physician builds less rapport with obese patients [51]. Indeed, the weight curve during the study period exhibited a natural deterioration in the control group rather than a stable weight. However, in this group, biochemical values remained stable during the 12-week observation period. 

### 4.2. Morphometric Parameters

Substantial weight loss was observed in the Dietplus^®^ subjects. All measurements, in particular impedancemetry, confirmed a reduction in abdominal obesity, with positive consequences on vital parameters, namely, a decrease in heart rate, hypertension, and cardiovascular risk [52,53]. Physical indices of comorbidity risk were also clearly improved. However, this study is limited by a relatively short-term follow-up. Hence, systematic annual monitoring of the 77 subjects who had completed the protocol is underway. 

### 4.3. Behavioral Parameters

The Dietplus^®^ program induced positive effects on the behavior of obese subjects, with improved QOL, Nutriscore, motivation to change lifestyle, and (discrete) improvement in physical activity. The necessity of adherence to a strict protocol is confirmed [46,54]. 

### 4.4. Metabolic Parameters

Several metabolic parameters were significantly improved over the 12-week study period, certainly in relation to the average 9 kg weight loss. 

#### 4.4.1. Glucose Metabolism

Insulin resistance was reduced with a lower HOMA index at the end of the study. Type II diabetes, rare in these subjects, became normalized for two participants. 

#### 4.4.2. Lipid Metabolism

Severe dyslipidemia, measured in 55/77 subjects at enrollment, was significantly improved. A significant benefit (31–79%) was observed in the plasma concentrations of different fractions except HDL cholesterol. These changes suggest reduced cardiovascular risk (transformation from phenotype A to B [55,56]). Supplementation of Omega-3s oils (mackerel, sardines, salmon, etc.) should be prescribed [57,58].

#### 4.4.3. Liver Function

A BMI > 30 predisposes individuals to non-alcoholic steatohepatitis (NASH) [59]. GGT is a reliable marker of NASH and cardiovascular risk and severity in subjects with metabolic syndrome [60]. Alcohol consumption was very limited in both cohorts. These results confirm the improvement of liver function and NASH after lifestyle improvement [61]. 

#### 4.4.4. Inflammatory Syndrome

The inflammatory syndrome of obesity, which perpetuates immune and metabolic disorders, improved. UsCRP levels significantly decreased at the end of the study period. PINI values also reflected this. Such improvement reflects reduced metabolic syndrome and underlying cardiovascular risk [62,63]. 

#### 4.4.5. 25-OH-Vitamin D

Although no causal relationship has been strongly identified [64,65], many studies have reported associations between vitamin D deficiency and obesity due to diluted liposoluble vitamin D in the increased fat mass [65]. Another factor responsible for the deficiency could be chronic low-grade inflammation in many subjects with metabolic syndrome, obesity, and diabetes [66,67]. 

25-OH-vitamin D deficiency is common and endemic in Belgium, as in other countries with low levels of winter sunlight. According to the European Food Safety Agency (EFSA) [68], a serum vitamin D level < 20 ng/mL (50 mmol/L) indicates deficiency. However, others, including the American Society of Endocrinology, consider that vitamin D levels should not be < 30 ng/mL (75 mmol/L) [69]. Nevertheless, a normal vitamin D status could improve the balance between pro- and anti-inflammatory cytokines, positively affecting insulin action and lipid metabolism. 

As a rule, these levels can be obtained in winter (the season most prone to deficiency) by taking PDFSs in doses adapted to age, state of health, sex, and body type [70,71]. Considering that this clinical study was started in December 2021, low plasma 25-OH-vitamin D levels during the first half of the study can be interpreted as a seasonal deficiency. However, the trends became more pronounced between the summer 2021 samples and the autumn controls. Several interpretations are plausible, including diet changes, exposure to light during outdoor activities, or melting of abdominal fat. Regardless, this favorable increase in 25-OH-vitamin D levels can rule out concerns [72,73]. 

#### 4.4.6. The Role of the Different Factors of the Dietplus^®^ Method 

The Dietplus^®^ method is based on three major actions: nutritional education, personalized coaching, and supply of PDFS supplementation. However, the program is considered holistic, making it difficult to determine the contributions made by each factor precisely. The benefits of personalized coaching are well documented in the literature [74,75]. Food rebalancing and technical dietary advice are also validated [76,77,78]. Meanwhile, reports concerning the effect of PDFSs on weight control are inconsistent. Several studies, including systematic reviews, report the absence of any evident effect elicited by PDFSs [79,80,81], whereas others provide scientific evidence [82,83], even if the limited effect of fat burners is denounced [84]. Nevertheless, the biological effects of certain PDFSs have been demonstrated for bilberry [85], cinnamon extract [86], green tea [87], artichoke leaf extract [88], chitosan [89], nopal, through gut microbiota adjustment [90], barberry [91] and phenylephrine [92]. Indeed, several researchers have denounced the lack of evidence-based data [93]. However, a placebo effect cannot be ruled out [94]. 

## 5. Conclusions

Healthcare systems are facing a global overweight and obesity pandemic. While many non-invasive methods lack scientific evaluation and evidence-based support, the present study provides evidence of a clear benefit for the Dietplus^®^ program. However, the impact of this study is limited by several constraints, including the absence of a double-blind controlled procedure and the concept of a totum program with different modes of action, excluding physical training [95]. Indeed, a substantial improvement in metabolic parameters and behavioral changes were observed that are likely to guarantee long-term weight control. Moreover, the contribution of the Dietplus^®^ program could be considered in terms of cost-effectiveness [96] within the panel of therapeutic approaches to weight control.

## Figures and Tables

**Table 1:** Analysis of Dietplus^®^ Abandonment (distinguished from study dropout).

Cause of Abandonment	Dietplus^®^ Group (*n* = 80/170)	Control Group (*n* = 12/30)
Lack of compliance (cure)	21 (26.2%)	5 (41.7%)
Medical exclusion criteria	12 (15.0%)	4 (33.4%)
Family constraints	12 (15.0%)	1 (8.3%)
Financial constraints	11 (13.7%)	1 (8.3%)
Logistic (Prof) incompatibility	10 (12.5%)	0 (0%)
Clinical study	6 (7.5%)	1 (8.3%)
Vegetarian	3 (3.8%)	0 (0%)
Psychological disorder	3 (3.8%)	0 (0%)
Family practice doctor refusal	2 (2.5%)	0 (0%)

**Table 2:** Anthropometric data of Dietplus^®^ subjects compared with controls.

Parameter	Dietplus^®^ Group (*n* = 170)	Control Group (*n* = 30)	*p*-Value
Age (y)	45 ± 12	45 ± 12	n.s.*
Sex ratio (f/m%)	89/11	89/11	n.s.
Weight (kg)	94.1 ± 14.2	100.0 ± 15.3	n.s.
Excess weight (%)	39.6 ± 4.0	43.5 ± 4.2	*p* < 0.05
BMI (kg/m^2^)	34.9 ± 3.7	36 ± 4	n.s.
Abd. Circumference (cm)	110 ± 132	109 ± 12.5	n.s.
Comorbidity %	46	62	*p* < 0.001
Dropout %	59	40	*p* < 0.05

* n.s.: not significant *p* > 0.05.

**Table 3:** Pretherapeutic scores in both groups.

Parameter	Dietplus^®^ Group (*n* = 77/170)	Control Group (*n* = 18/30)	*p*-Value
Quality of Life (score/60)	34.4 ± 6.0	29 ± 5.4	n.s.
Nutriscore (score/100)	43.5 ± 4.2	59 ± 5.7	*p* < 0.05
Physical Activity (score/100)	40.9 ± 9.9	40 ± 9.3	n.s.
Prochaska Di Clemente Scale	7.8 ± 4.1	4.0 ± 1.3	*p* < 0.05

n.s.: not significant *p* > 0.05.

**Table 4:** Evolution of scores in the control group during the observation period.

Parameter	At Enrollment (*n* = 18)	12-Week Work-up (*n* = 18)	*p*-Value
Quality of Life (score/60)	29.0 ± 5.4	29.9 ± 5.3	n.s.
Nutriscore (score/100)	58.9 ± 5.7	61.5 ± 7.4	n.s.
Physical Activity (score/100)	39.5 ± 9.6	40.8 ±9.3	n.s.
Prochaska Di Clemente Scale	4.0 ± 1.3	7.0 ± 4.5	*p* < 0.05

n.s.: not significant *p* > 0.05.

**Table 5:** Evolution of body composition by Impedancemetry.

Parameter	At Enrollment (*n* = 77)	12-Week Work-Up (*n* = 77)	*p*-Value
FAT %	42.45 ± 10.11	35.06 ±9.40	*p* < 0.001
WATER %	38.79 ± 7.44	37.65 ±7.13	n.s.
MUSCLE %	29.43 ± 6.10	28.52 ± 5.83	n.s.

n.s.: not significant *p* > 0.05.

**Table 6:** Effect of the Dietplus^®^ method on subject motivation.

	Precontemplation	Contemplation	Determination	Action
**Dietplus^®^ Subjects’** Score at enrollment (*n* = 74)	38	32	4	0
**Cure and study completed.** (*n* = 74)	12	43	18	1

**Table 7:** Changes in glucose metabolism.

Parameter	At Enrollment	12-Week Work-Up	*p*-Value
High Glucose concentration	7/77 (10.0%)	5/77 (6.5%)	n.s.
Insulin increased	11/49 (22.4%)	3/49 (6.1%)	*p* < 0.05.
HOMA > 2.26	26/49 (53.1%)	11/49 (22%)	*p* < 0.01
QUICKI	28/49 (57.1%)	15/49 (30.6%)	*p* < 0.01

n.s.: not significant *p* > 0.05.

**Table 8:** Effects of the cure on lipid profile.

Parameter	At Enrollment (*n* = 77)	12-Week Work-Up (*n* = 77)	*p*-Value
Total cholesterol (mg/dL)	202.8 ± 47.1	190.0 ± 43.7	*p* < 0.001
Triglycerides (mg/dL)	135.5 ± 68.3	118.1 ± 52.7	*p* < 0.005
HDL (mg/dL)	54.0 ± 15.4	52.9 ± 12.6	n.s.
LDL (mg/dL)	121.6 ± 40.4	113.5 ± 38.7	*p* < 0.001
VLDL (mg/dL)	27.2 ± 13.7	23.6 ± 10.5	*p* < 0.005
computed non-HDL (mg/dL)	149.8 ± 45.8	137.1 ± 42.8	*p* < 0.001
Phenotype A Ln (Tg/HDL)	45/77 (58%)	28/77 (36%)	*p* < 0.01

n.s.: not significant *p* > 0.05.

**Table 9:** Changes in the inflammatory syndrome.

Parameter	At Enrollment (*n* = 77)	12-Week Work-Up (*n* = 77)	*p*-Value
Fibrinogen (mg/dL)	355.57 ± 75.99	357.92 ± 66.19	n.s.
CRP (mg/dL)	6.42 ± 6.67	5.70 ± 5.89	*p* < 0.001
usCRP (mg/dL)	2.55 ± 1.98	2.30 ± 1.84	*p* < 0.001
Orosomucoid (g/L)	0.89 ± 0.19	0.84 ± 0.20	*p* < 0.001
PINI 1 index	0.56 ± 0.65	0.46 ± 0.53	*p* < 0.001
PINI 2 index	0.35 ± 0.34	0.30 ± 0.33	*p* < 0.05

n.s.: not significant *p* > 0.05.

## Data Availability

Original tables and Supplementary Materials presented by tables are available online at www.gastrospace.com/nutrients/, accessed on 15 May 2023.

## Supplementary Materials

The following supporting information can be downloaded at: www.gastrospace.com/nutrients/.
